# Guideline-concordant treatment among adolescents and young adults with acute lymphoblastic leukemia

**DOI:** 10.1093/jncics/pkaf033

**Published:** 2025-04-16

**Authors:** Julie A Wolfson, Allison C Grimes, Michelle M Nuño, Subhash Ramakrishnan, David S Dickens, Michael E Roth, Wendy Woods, Kandice S Adams, Tawa Alabi, Melissa Beauchemin, Jennifer M Levine, Michele Scialla, Koh B Boayue, Charlotte L Kerber, Olivia Ponce, Sarah Vargas, George J Chang, Wendy Stock, Dawn Hershman, Emily Curran, Anjali Advani, Kristen O’Dwyer, Selina Luger, Jane Jijun Liu, David R Freyer, Lillian Sung, Susan K Parsons

**Affiliations:** Division of Pediatric Hematology-Oncology, University of Alabama at Birmingham, Birmingham, AL, United States; Institute for Cancer Outcomes and Survivorship, University of Alabama at Birmingham, Birmingham, AL, United States; Division of Pediatric Hematology-Oncology, University of Texas Health Science Center San Antonio, San Antonio, TX, United States; Children’s Oncology Group, Monrovia, CA, United States; Department of Population and Public Health Sciences, University of Southern California, Los Angeles, CA, United States; Children’s Oncology Group, Monrovia, CA, United States; Division of Pediatric Hematology-Oncology, University of Iowa, Iowa City, IA, United States; Division of Pediatric Oncology, MD Anderson Cancer Center, Houston, TX, United States; Division of Pediatric Hematology-Oncology, Blank Children’s Hospital, Des Moines, IA, United States; Institute for Cancer Outcomes and Survivorship, University of Alabama at Birmingham, Birmingham, AL, United States; Institute for Cancer Outcomes and Survivorship, University of Alabama at Birmingham, Birmingham, AL, United States; School of Nursing, Columbia University Irving Medical Center, New York, NY, United States; Center for Cancer and Blood Disorders, Children’s National Medical Center, Washington, DC, United States; Center for Healthcare Delivery Science, Nemours Children’s Hospital, Delaware, Wilmington, DE, United States; Division of Pediatric Hematology-Oncology, University of New Mexico Cancer Center, Albuquerque, NM, United States; Department of Public Health Sciences, University of California, Davis, Davis, CA, United States; Children’s Oncology Group, Monrovia, CA, United States; Children’s Oncology Group, Monrovia, CA, United States; Department of Colon and Rectal Surgery and Department of Health Services Research, The University of Texas, MD Anderson Cancer Center, Houston, TX, United States; Division of Hematology-Oncology, University of Chicago Medicine, Chicago, IL, United States; Division of Hematology-Oncology, Columbia University, New York, NY, United States; Division of Hematology-Oncology, University of Cincinnati, Cincinnati, OH, United States; Division of Hematology-Oncology, Cleveland Clinic, Taussig Cancer Institute, Cleveland, OH, USA; Division of Hematology-Oncology, University of Rochester, Wilmot Cancer Institute, Rochester, NY, United States; Division of Hematology-Oncology, University of Pennsylvania, Philadelphia, PA, USA; Heartland National Cancer Institute Community Oncology Research Program, Illinois CancerCare, Peoria, IL, United States; Division of Pediatric Hematology-Oncology, Children’s Hospital Los Angeles, Los Angeles, CA, United States; Division of Haematology/Oncology, The Hospital for Sick Children, Toronto, ON, Canada; Division of Hematology/Oncology and Institute for Clinical Research and Health Policy Studies, Tufts Medical Center, Boston, MA, United States

## Abstract

**Background:**

Individuals diagnosed with acute lymphoblastic leukemia (ALL) between adolescents and young adults aged 15-39 years face poor survival and unique challenges. We evaluated facility-level factors and guideline-concordant care among adolescents and young adults with ALL at National Cancer Institute Community Oncology Research Program (NCORP) practices.

**Methods:**

We assembled a retrospective cohort of adolescents and young adults aged 15-39 years with ALL treated at participating NCORPs between 2012 and 2016. NCORPs abstracted patient data and completed facility-level questionnaires for each clinical facility (study-defined criteria). The central review committee adjudicated whether treatment was concordant with adolescent and young adult–specific National Comprehensive Cancer Network ALL guidelines (ie, pediatric-inspired therapy or clinical trial). Guideline-concordant care was described by age, facility model (adult/internal medicine, pediatric, mixed [pediatric services within a general hospital]), and average annual adolescents and young adult ALL volume. Generalized linear mixed effects models estimated the odds of guideline-concordant care.

**Results:**

Adolescents and young adults receiving guideline-concordant care were younger (n = 196; median = 19.5 years) than those who did not (n = 31; median = 32.1 years). Guideline-concordant care was observed in many adolescents and young adults aged 22-39 years (68.8%), and nearly universal in those aged 15-21 years. In multivariable analyses, adolescents and young adults at adult/internal medicine clinical facilities had lower odds of guideline-concordant care (odds ratio = 0.02, 95% confidence interval = 0.0 to 0.18); there was no statistically significant association between annual adolescent and young ALL volume and receiving guideline-concordant care. Guideline-concordant care was observed more often in adult/internal medicine and/or mixed clinical facilities with communication between adult or pediatric counterparts, adolescents and young adult ALL clinical pathways, and/or adolescent and young adult–specific meetings.

**Conclusion:**

Guideline-concordant care among adolescents and young adults with ALL (specifically pediatric-inspired therapy) at NCORPs is associated with facility model (adult/internal medicine) but not adolescent and young adult ALL volume. Strategies to improve guideline-concordant care could include facilitating communication and clinical pathways at adult/internal medicine clinical facilities treating adolescent and young adult ALL.

## Introduction

Individuals diagnosed with cancer between the ages of 15 and 39 years face disparate survival trends and have distinct psychosocial needs, along with a unique interface with the health-care system. Thus, the National Cancer Institute (NCI) prioritizes work related to adolescent and young adult cancer.^1-3^ Although in recent decades outcomes have improved for adolescents and young adults as a whole,^4^ survival among those with acute lymphoblastic leukemia (ALL) remains inferior to that of children (5-year relative survival: 0-14 years = 92%, 15-39 years = 63%)^4^ and decreases with age.^4-6^

In adolescent and young adult ALL, less attention has focused on care delivery than biologic and clinical aspects.^7^ Outcome differences differ by location of care,^8-10^ but prior hypothesis-generating work used registry-level data without structure- or process-level details. ALL is unique in that there are robust adolescent and young adult–specific national treatment guidelines. In 2012, the National Comprehensive Cancer Network (NCCN) began disseminating ALL guidelines with age-specific recommendations (15-39 years vs 40 years and older).^11^ With evidence of superior adolescent and young adult survival with pediatric-inspired vs adult-type regimens,^6^ NCCN guidelines from 2012 to 2016 recommended that treatment for adolescents and young adults with Philadelphia-chromosome negative (Ph-neg) ALL and T-cell ALL (T-ALL) include pediatric-inspired multi-agent chemotherapy or a clinical trial.^12^ Although data suggest the minority of adolescents and young adults are treated in this way, these findings were limited to registry-level^13^ and National Clinical Trial Network data.^14^

With the majority of adolescent and young adult ALL treated in community facilities rather than academic facilities,^8^^,^^15^ the optimal place to engage in facility-level adolescent and young adult research is a community-based practice. The NCI Community Oncology Research Program (NCORP)^16-18^ aims to increase access to cancer clinical trials for patients in their communities thus providing an ideal setting for evaluating the quality-of-care delivery in adolescents and young adults. Our study (ACCL16N1CD [NCT03204916 on clinicaltrials.gov]) sought to assess NCCN guideline concordance of adolescent and young adult ALL treatment at NCORPs, aiming to elucidate gaps in equitable care.

## Methods

### Facility participation

Facility recruitment and classification has been described elsewhere.^19^ Briefly, participating practices were NCORP members and treated at least 1 individual aged 15-39 years with B-cell or T-cell ALL between January 2012 and December 2016. This Intergroup NCORP Cancer Care Delivery Research study was led by Children’s Oncology Group and its NCORP research base, with study champions from adult NCORP research bases (SWOG Cancer Network, ECOG-ACRIN Cancer Research Group, Alliance for Clinical Trials in Oncology).^20^ Institutions activated the study via their preferred NCORP research base using clinical trials and evaluation program research identifiers. Inasmuch as clinical care within NCORPs is delivered across an array of individual practices that do not necessarily correlate with research identifiers, previously published concrete criteria (including separate admitting privileges, requirement to transfer between facilities)^19^ distinguished study-defined independent clinical entities (or practices), termed *clinical facilities*, rather than using Cancer Therapy Evaluation Program identifiers.

### Facility classification and characteristics

Based on a series of study-specific questions regarding services, clinical facilities were classified according to facility model (pediatric, adult/internal medicine, mixed [pediatric services embedded within a general health-care facility]).^19^ We focused on the following structure- and process-level characteristics captured via questionnaire:^19^ annual adolescent and young adult ALL volume; facility model; and use of an adolescent and young adult ALL clinical pathway (defined as a clear algorithm or clinical pathway for determining cancer care).^21^^,^^22^ Clinical facilities were classified as having the potential to communicate when they were affiliated with at least 1 cross-age (pediatric or adult) counterpart (if applicable). Further, if cross-age counterparts had contact (at any frequency), the clinical facility was classified as having actual communication. A combined measure reflecting contact included attending each other’s tumor boards, any adolescent and young adult–specific meetings (programmatic, research, tumor board, journal club), or individual discussions; prevalence of the discrete measures was described previously.^19^

### Patients

Eligible patients were aged 15-39 years at ALL diagnosis and treated at a participating NCORP between 2012 and 2016. Adolescents and young adults with B-cell ALL (Ph-neg and Ph-pos) and T-ALL were included.

Institutions could identify eligible patients using cancer registry or billing data; most used local cancer registries and *International Classification of Diseases for Oncology 3rd edition* diagnostic codes. After receiving de-identified lists of eligible patients from each clinical facility across NCORPs, Children’s Oncology Group study statisticians randomly sampled 270 patients for chart review, stratified by age group (15-17 years, 18-21 years, 22-39 years) and clinical facility.

For sampled patients, sites submitted sociodemographic (age, race, ethnicity, insurance), clinical (immunophenotype, cytogenetics, minimal residual disease), treatment (regimen, clinical trial enrollment, dates), and physician (subspecialty training) data with supporting documentation (provider, pharmacy, nursing); these were reviewed by the University of Alabama at Birmingham (UAB) Coordinating Center.

### Guideline concordance: Definition and central review

During the study period, NCCN ALL guidelines recommended^11^^,^^23^ treatment of newly diagnosed patients aged 15-39 years with Ph-neg ALL and T-ALL with pediatric-inspired therapy or a clinical trial, specifying which regimens were pediatric-inspired vs designated for individuals aged 40 years and older. For newly diagnosed individuals aged 15-39 years with Ph-pos ALL, the guidelines recommended treatment with multi-agent chemotherapy and a tyrosine kinase inhibitor, providing a list of recommended regimens. Using these definitions of guideline concordance, the study team standardized an adjudication template (available on request; approved by the Central Review Committee [JAW, ACG, DD, WW, MER]). The UAB Coordinating Center populated the template using data sites submitted electronically and any supporting documentation. Panels consisted of at least 3 reviewers, with the study chair present for all; after cross-referencing guideline versions and regimen details, the panel voted on elements of guideline concordant care (ie, clinical trial enrollment, treatment approach) for each treatment phase (induction and postinduction therapy). Because of varying terminology across treatment regimens, postinduction therapy was used to represent all therapy following induction. Treatment was considered guideline-concordant for a phase if the patient enrolled on a trial or received a treatment on the guideline-concordant list (or successor regimen). Patients were considered to have received guideline-concordant care if both induction and postinduction therapy were concordant. All determinations were unanimous.

### Analysis

Logistic mixed effects models were used to model the odds of delivering guideline-concordant care; we report estimated odds ratios (ORs) and 95% confidence intervals (CIs). Random intercepts were used to account for within-site correlation, and site and patient characteristics were modeled using fixed effects. This study was not powered to address patient-level factors such as sociodemographic characteristics, however, exploratory descriptive statistics were calculated, including median, interquartile range (IQR), and proportions. Because of the descriptive nature of the portion of the study surrounding process-level facility characteristics, formal hypothesis tests were not conducted, and we instead present descriptive statistics by facility model and size. The Pediatric Central Institutional Review Board (IRB; Rockville, MD, USA) approved this study, including a consent waiver. Local IRB approval was in accordance with institutional policies; UAB IRB approved the Coordinating Center Protocol. A total of 270 patients were randomly sampled from eligible patients. This provided for a 20% buffer of ineligible patients to achieve the target sample (n = 225) and ensured adequate representation by age, supporting the precision for analysis. A sample size of 225 was selected at the design stage as it would provide satisfactory precision if approximately 48% of patients received guideline-concordant care. This estimate was based on previous studies reporting that approximately 30% of medical oncologists and 80% of pediatric oncologists would recommend treatment according to NCCN guidelines. R version 4.2.2 and a data cutoff date of March 3, 2024, were used for analyses.

## Results

### Patients

After review, 41 of 270 sampled patients were ineligible, leaving 229 eligible patients (Figure S1). The panel determined that 2 did not have a documented treatment plan; thus guideline-concordant care was assessed in 227 patients. The cohort was majority male, with each age group representing roughly one-third of the sample (Table 1). Most patients were non-Hispanic White (35.7%) or Hispanic or Latino (32.6%), followed by Black or African American (7.5%), which was consistent across age groups. Public insurance was observed more commonly in younger (15-17 years = 45.0%, 18-21 years = 44.8%) than older (22-39 years = 37.5%) adolescents and young adults. Much of the cohort across age groups had Ph-neg B-ALL (n = 160, 70.5%). Although T-ALL (n = 41, 18.1%) and Ph-pos B-ALL (n = 26, 11.5%) comprised less of the total cohort, T-ALL was observed more often in younger (15-17 years  = 23.8%; 18-21 years = 19.4%; 22-39 years = 11.3%) and Ph-pos ALL in older (15-17 years = 10.0%; 18-21 years = 6.0%; 22-39 years = 17.5%) adolescents and young adults. Adolescents and young adults with Down syndrome represented 2% of the cohort.

**Table 1.** Patient characteristics (by age group)

Characteristics	All patients, No. (%) (n = 227)	Aged 15-17 y, No. (%) (n = 80)	Aged 18-21 y, No. (%) (n = 67)	Aged 22-39 y, No. (%) (n = 80)
Sex				
Female	66 (29.1)	21 (26.3)	16 (23.9)	29 (36.3)
Male	134 (59.0)	55 (68.8)	40 (59.7)	39 (48.8)
Unknown	27 (11.9)	4 (5.0)	11 (16.4)	12 (15.0)
Race and ethnicity				
Black, African American	17 (7.5)	5 (6.3)	6 (9.0)	6 (7.5)
Hispanic, Latino	74 (32.6)	30 (37.5)	16 (23.9)	28 (35.0)
Non-Hispanic White	81 (35.7)	28 (35.0)	23 (34.3)	30 (37.5)
Other, unknown^a^	55 (24.2)	17 (21.3)	22 (32.8)	16 (20.0)
Insurance				
Public	96 (42.3)	36 (45.0)	30 (44.8)	30 (37.5)
Private	108 (47.6)	40 (50.0)	32 (47.8)	36 (45.0)
None	15 (6.6)	4 (5.0)	4 (6.0)	7 (8.8)
Other, unknown	8 (3.5)	0 (0)	1 (1.5)	7 (8.8)
Diagnosis				
B-cell ALL: Philadelphia chromosome negative	160 (70.5)	53 (66.3)	50 (74.6)	57 (71.3)
T-cell ALL	41 (18.1)	19 (23.8)	13 (19.4)	9 (11.3)
B-cell ALL: Philadelphia chromosome positive	26 (11.5)	8 (10.0)	4 (6.0)	14 (17.5)
Down syndrome				
Yes	5 (2.2)	1 (1.3)	3 (4.5)	1 (1.3)
Clinical facility model				
Pediatric	67 (29.5)	46 (57.5)	18 (26.9)	3 (3.8)
Adult/internal medicine	69 (30.4)	0 (0)	20 (29.9)	49 (61.3)
Mixed	91 (40.1)	34 (42.5)	29 (43.0)	28 (35.0)
Clinical facility volume: adolescent and young adult ALL patients				
<5 patients/year	70 (30.8)	29 (36.3)	20 (29.9)	21 (26.3)
5-10 patients/year	79 (34.8)	28 (35.0)	23 (34.3)	28 (35.0)
>10 patients/year	57 (25.1)	20 (25.0)	18 (26.9)	19(23.8)
Missing	21 (9.3)	3 (3.8)	6 (9.0)	12 (15.0)

Abbreviation: ALL = acute lymphoblastic leukemia.

a Other race includes Asian and more than once race.

### Clinical facilities

Among 61 clinical facilities treating these adolescents and young adults, 41% operated within an adult/internal medicine model of care, 36% within a mixed model, and 23% within a pediatric model. Few of the 57 clinical facilities reporting their adolescent and young adult ALL volume saw large annual volumes of adolescent and young adult ALL (<5 = 36.8%, 5-10 = 42.1%, 10-25 = 14.0%, >25 = 7.0%) (Figure S2). Volume is categorized in 3 groups in the analysis in light of the distribution (<5, 5-10, ≥10). Adolescents and young adults aged 15-17 years were treated at pediatric (58%) or mixed (43%) clinical facilities; individuals aged 18-21 years were distributed across pediatric (27%), adult/internal medicine (30%), and mixed (43%) clinical facilities; and those aged 22-39 years were observed mostly at adult/internal medicine clinical facilities (pediatric = 4%, adult/internal medicine = 61%, mixed = 35%) (Table 1).

### Guideline-concordant treatment

**Figure 1.**
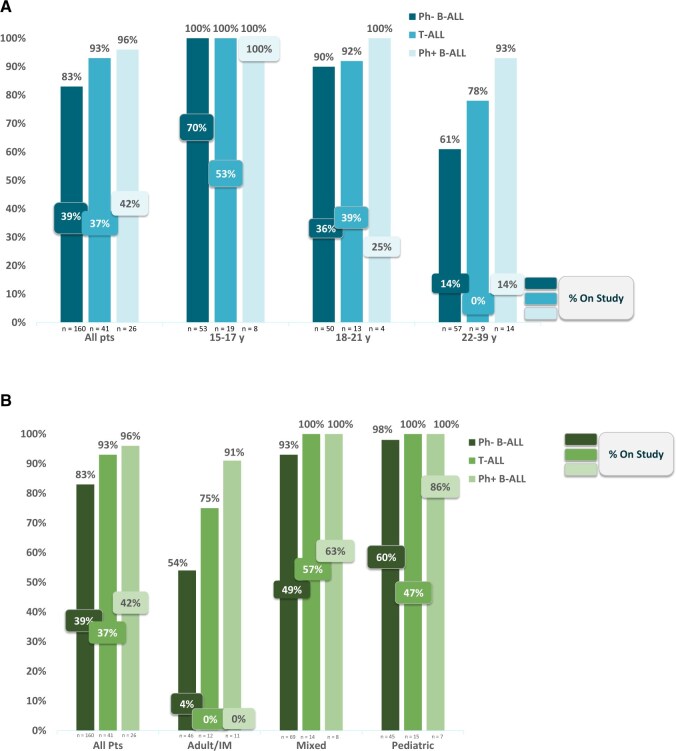
Guideline-concordant treatment by age group and facility model. Figure 1 presents the rate of guideline-concordant care among adolescents and young adults by (**A**) age group and (**B**) facility model. Each panel presents a series of bars that represent Philadelphia-chromosome negative (Ph-) and positive (Ph+) B-cell acute lymphoblastic leukemia (ALL), and T-cell ALL. All patients who received guideline-concordant care received a guideline-concordant regimen (Ph- and T-ALL = pediatric-inspired therapy; Ph+ = multi-agent plus tyrosine kinase inhibitor). The total number of each subgroup is represented on the *x*-axis. Abbreviations: IM = internal medicine; pts = patients.

The majority (n = 196, 86%) of patients received guideline-concordant care. All adolescents and young adults with guideline-concordant care received a recommended regimen (Ph-neg = pediatric inspired, Ph-pos = multi-agent chemotherapy with tyrosine kinase inhibitor); among 10 guideline-concordant care patients aged 18 years and older, a tyrosine kinase inhibitor was coupled with a pediatric-inspired regimen in half (AALL1131, AALL0232), and an adult-style regimen (GRAAPH-2005, ALL-2, Larson) in half. Smaller proportions of adolescents and young adults were observed to be treated on a clinical trial (Ph-neg = 39%, T-ALL = 37%, Ph-pos = 42%), with younger adolescents and young adults noted to be on trials more often than older adolescents and young adults (Figure 1, A and B). Across age groups, trial enrollment was observed less often at adult/internal medicine clinical facilities and more often at pediatric and mixed clinical facilities (Figure 1, B). Adolescents and young adults treated by pediatric oncologists were noted to be enrolled on clinical trials more often than those treated by adult oncologists (Table S1).

#### Age, facility model, and annual volume of adolescent and young adult ALL

Adolescents and young adults who received guideline-concordant care were observed to be younger (median = 19.5 years, IQR = 16.6-27.3 years) than those who did not (median = 32.1 years, IQR = 24.0-34.6 years). Although the majority of those aged 22-39 years received guideline-concordant care (Ph-neg = 61%, T-ALL = 78%, Ph-pos = 93%), guideline-concordant care was observed among them less often than other age groups (15-17 years = 100% across subtypes; 18-21 years: Ph-neg = 90%, T-ALL = 92%, Ph-pos = 100%) (Figure 1, A).

**Figure 2.**
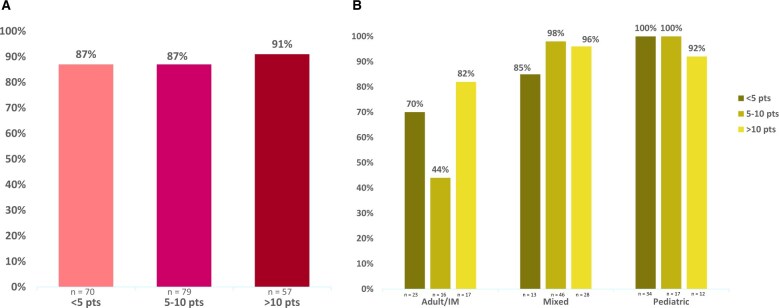
Guideline-concordant treatment by annual volume of adolescent and young adult acute lymphoblastic leukemia (ALL). Figure 2 presents the rate of guideline-concordant care among adolescents and young adults by (**A**) annual volume of adolescent and young adult ALL and (**B**) facility model and volume. Of note, not all clinical facilities responded to the question regarding average annual ALL volume. Findings represent the proportion of patients with a known clinical facility volume. The total number of each subgroup is represented on the *x*-axis. Abbreviations: IM = internal medicine; pts = patients.

Considering facility model alone, fewer adolescents and young adults (albeit the majority) at adult/internal medicine clinical facilities (Ph-neg = 54%, T-ALL = 75%, Ph-pos = 91%) than at mixed (Ph-neg = 93%, T-ALL = 100%, Ph-pos = 100%) or pediatric (Ph-neg = 98%, all T-ALL = 100%, Ph-pos = 100%) clinical facilities were observed to receive guideline-concordant care (Figure 1, B). Considering only annual adolescent and young adult ALL volume, a similar percentage of adolescents and young adults were noted to receive guideline-concordant care across clinical facilities regardless of volume (<5 = 87%, 5-10 = 87%, >10 = 91%) (Figure 2, A). When considering facility model and adolescent and young adult ALL volume, the pattern of guideline-concordant care was observed to be inconsistent at adult/internal medicine clinical facilities by volume (<5 patients per year = 70%, 5-10 patients per year = 44%, >10 patients per year = 82%) but consistent at mixed (<5 patients per year = 85%, 5-10 patients per year = 98%, >10 patients per year = 96%) and pediatric (<5 patients per year = 100%, 5-10 patients per year = 100%, >10 patients per year = 92%) clinical facilities regardless of volume (Figure 2, B).

Fewer adolescents and young adults treated by adult (Ph-neg = 62%, T-ALL = 79%, Ph-pos = 93%) than pediatric (Ph-neg = 99%, all T-ALL and Ph-pos) oncologists were observed to receive guideline-concordant care (Table S1).

#### Odds of guideline-concordant care delivery

In univariable analyses, adolescents and young adults at adult clinical facilities had 97% lower odds (OR = 0.03, 95% CI = 0.01 to 0.18; referent: pediatrics and mixed clinical facilities) of receiving guideline-concordant care. Multivariable analysis adjusting for adolescent and young adult ALL volume found similar results (OR = 0.02, 95% CI = 0.00 to 0.18) (Table 2). The association between annual volume of adolescent and young adult ALL and guideline-concordant care was not statistically significant (Table S2).

**Table 2.** Odds of delivery of guideline-concordant treatment^a^

Model	Variable	Level	Odds Ratio (95% Confidence Interval)
Univariable	Facility model^b^	Pediatric plus mixed	(referent)
		Adult/internal medicine	**0.03 (0.01 to 0.18)**
	Facility volume^c^^,^^d^	<5 patients	(referent)
		5-10 patients	0.87 (0.01 to 54.58)
		>10 patients	2.00 (0.01 to 438.72)
Multivariable^d^	Facility model	Pediatric plus mixed	(referent)
		Adult/internal medicine	**0.02 (0.00 to 0.18)**
	Facility volume	<5 patients	(referent)
		5-10 patients	0.61 (0.09 to 4.17)
		>10 patients	0.56 (0.05 to 6.14)

Abbreviation: CI = confidence interval.

a Of note, participating clinical facilities provided facility model and volume in the site questionnaire.

b Clinical facility classification as delivering care in a pediatric model, adult/internal medicine model, or a mixed model (pediatric services embedded within a general hospital).

c Average volume of adolescents and young adults (aged 15-39 years) with acute lymphoblastic leukemia each year.

d Complete-case analysis was conducted because of patients missing adolescent and young adult acute lymphoblastic leukemia volume.

#### Adolescent and young adult–specific process characteristics

**Figure 3.**
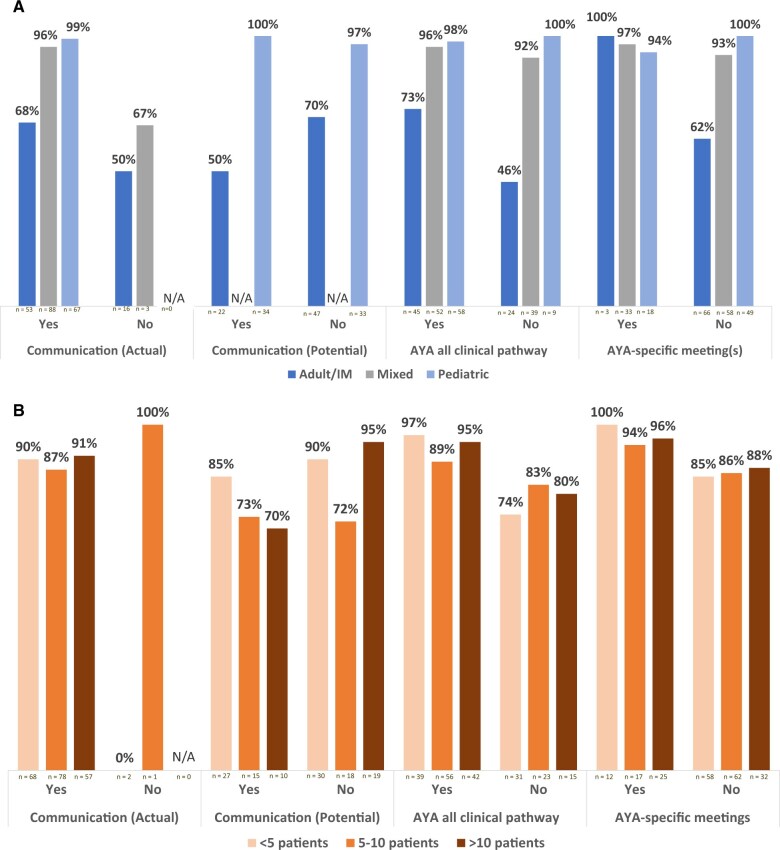
Adolescent and young adult–specific process characteristics and guideline-concordant therapy by facility model and annual adolescent and young adult volume. Figure 3 presents the rate of guideline-concordant care among adolescents and young adults by the presence of discrete process characteristics and (**A**) facility model and (**B**) annual volume of adolescent and young adult acute lymphoblastic leukemia. The total number of each subgroup is represented on the *x*-axis. Abbreviations: ALL = acute lymphoblastic leukemia; AYA = adolescent and young adult; IM = internal medicine; N/A = not applicable.

At adult/internal medicine clinical facilities, guideline-concordant care rates were observed to be higher when there was communication between cross-age counterparts (68% vs 50%), an adolescent and young adult ALL clinical pathway (73% vs 46%), and/or adolescent and young adult–specific meetings (100% vs 62%) and lower at adult/internal medicine clinical facilities with the potential for communication (50% vs 70%) (Figure 3, A). At mixed clinical facilities, guideline-concordant care rates were noted to be higher when there was communication with cross-age counterparts (96% vs 67%), although in the same range with or without adolescent and young adult ALL clinical pathways (96% vs 92%) or adolescent and young adult–specific meetings (97% vs 93%). At pediatric clinical facilities, nearly all adolescents and young adults were observed to receive guideline-concordant care regardless of actual or potential communication, adolescent and young adult ALL clinical pathways, and/or adolescent and young adult–specific meetings.

Guideline-concordant care was observed to be higher among clinical facilities with adolescent and young adult ALL clinical pathways (<5 patients per year = 97% vs 74%; 5-10 patients per year = 89% vs 83%; >10 patients per year = 95% vs 80%) and adolescent and young adult–specific meetings (<5 patients per year = 100% vs 85%; 5-10 patients per year = 94% vs 86%; >10 patients per year = 96% vs 88%) than among clinical facilities without these characteristics (Figure 3, B). Most patients at clinical facilities with cross-age communication received guideline-concordant care; very few patients receiving guideline-concordant care were at clinical facilities without communication (n = 3).

#### Sociodemographics

Guideline-concordant care was noted to be similar across racial and ethnic categories: non-Hispanic White (86%), Hispanic and Latino (88%), and all others (African American, Asian, and >1 race: 86%). Adolescents and young adults were observed to receive guideline-concordant care more often if publicly (91%) insured, followed by private (86%) or no (77%) insurance (Table 3). When stratified by age, guideline-concordant care rates were observed to be similar to age- and race- and ethnicity-specific findings, with guideline-concordant care in all non-Hispanic White and non-White individuals aged 15-17 years, most aged 18-21 years (non-Hispanic White = 87%, non-White = 93%) and fewer aged 22-39 years (non-Hispanic White = 73%, non-White = 70%) (Table S3).

**Table 3.** Sociodemographics^a^ and guideline-concordant treatment

Patient characteristics	All patients [N]	Guideline concordant [n (%)]
Race and ethnicity		
All others^b^	36	31 (86.1%)
Hispanic, Latino	74	65 (87.8%)
Non-Hispanic White	81	70 (86.4%)
Unknown	36	30 (83.3%)
Insurance		
Public	96	87 (90.6%)
Private	108	93 (86.1%)
Uninsured, other	17	13 (76.5%)
Unknown	6	3 (50.0%)

a Sociodemographic characteristics abstracted by sites from medical record.

b All others includes Asian, Black or African American, more than 1 race.

## Discussion

Among adolescents and young adults with ALL treated at NCORPs, we identified clinically relevant differences in guideline-concordant care by age and location of care. Adolescents and young adults saw 98% lower odds of guideline-concordant care when treated at adult/internal medicine clinical facilities than at pediatric or mixed clinical facilities, after adjusting for adolescent and young adult ALL volume. Considering facility-level characteristics, we observed a lower rate of guideline-concordant care at clinical facilities without adolescent and young adult ALL-specific clinical pathways, communication between pediatric/adult counterparts, and/or adolescent and young adult–specific meetings. These findings provide an in-depth system-level examination of care delivery among adolescents and young adults with ALL.

Although the majority of adolescents and young adults aged 22-39 years received guideline-concordant care, more than 30% had non-guideline-concordant care, standing in contrast to other age groups, just as adult/internal medicine clinical facilities (where >35% of adolescents and young adults received nonguideline-concordant care) stand in contrast to pediatric or mixed clinical facilities. Most patients who received nonguideline-concordant care were older than 21 years of age and under the care of adult oncology. Each component of guideline-concordant care (treatment with pediatric-inspired therapy and/or on a clinical trial) faces unique implementation challenges.

All adolescents and young adults treated with guideline-concordant care received recommended regimens (Ph-neg or T-ALL = pediatric inspired, Ph-pos = multi-agent plus tyrosine kinase inhibitor). Nonguideline-concordant care was noted across only 14% of adolescents and young adults but 32% of those aged 22-39 years; however, 17% of Ph-pos adolescents and young adults received nonguideline-concordant care regimens in contrast to nearly 7% of Ph-neg and 4% of T-ALL patients. The higher use of guideline-concordant care regimens in Ph-pos ALL may reflect that guideline’s flexibility to use an adult-style regimen, which is familiar to the adult/internal medicine and providers, whereas the pediatric-inspired regimens required for Ph-neg and T-ALL are less familiar to adult systems and teams. Pediatric-inspired regimens are complex and multiphasic and delivered mostly outpatient. Experts comment that patient volume in adult oncology is not commensurate with the intricate needs of these regimens, including strong clinic infrastructure necessary for patient support and a crucial medication (asparaginase), which is rarely used in common adult cancers and has unique toxicities.^6^^,^^24^^,^^25^ Although some may surmise from these data that adolescents and young adults should be seen at pediatric or mixed model clinical facilities (because of higher guideline-concordant care rates), this is a pragmatic challenge; pediatric-inspired regimens are long (>2 years) and require substantial outpatient care, which may be challenging (social support, transportation, financial barriers) if a patient must travel for care.

The clinical trial enrollment component of guideline-concordant care varied. Although low clinical trial enrollment among adolescents and young adults (vs children) is well documented, proposed barriers to enrollment rely on provider-level qualitative studies or network-level studies without granular facility-level detail.^26-29^ Further work in the context of available trials is necessary to understand relevant barriers (and is under way).^28^

Because pediatric-inspired therapy was part of all documented guideline-concordant care, our findings specifically help understand delivery of this treatment (rather than guideline-concordant care as a concept). In addition, our study evaluated health-care–level factors, identifying that facility model alone—not volume—was associated with delivering guideline-concordant care in this population, even when stratified by model; this stands in contrast to expert hypotheses surrounding the low rate of pediatric-inspired therapy,^13^ often focusing on physician-level barriers, such as experience or bandwidth.^6^^,^^25^ Quality measures in health care often involve practice volume, likely because much of that work is in procedural specialties (surgery,^30-34^ neonatology^35^^,^^36^); thus, it is particularly relevant for high-risk surgeries performed at low frequencies^37^^,^^38^ that require procedural skill.^39^ In our study, volume was not associated with guideline-concordant care. In oncology, volume is a recognized proxy measure for other aspects of care delivery, including characteristics related to health-care process and/or physician factors.^37^^,^^38^ This prompts the question: What facility-level characteristics may facilitate guideline-concordant care (specifically pediatric-inspired therapy) in this population, especially at adult/internal medicine clinical facilities?

Unique process-level characteristics surrounding physician and staff communication (communication between cross-age teams; adolescent and young adult–specific meetings) and clinical decision tools (such as an adolescent and young adult ALL clinical pathway) were reported at adult/internal medicine clinical facilities observed to use pediatric-inspired therapy more often. Across models and sizes of clinical facilities, we observed more pediatric-inspired therapy at clinical facilities with adolescent and young adult–specific meetings and clinical pathways. It is logical to consider strategies to enhance communication between specialists (across physical and esoteric bridges) and clinical decision tools (for practice standards) to facilitate use of a pediatric-inspired regimen in adult oncology.

Although there is room for improvement, it is encouraging that the majority of patients across NCORPs received recommended pediatric-inspired therapy. In regional population-level data from a similar time frame (including, but not limited to, NCORPs), pediatric-inspired regimens were given to small proportions of adolescents and young adults treated by adult oncologists outside pediatric-only centers (21%-31%), older adolescents and young adults (aged 19 years and older = 21%), and/or at low-volume sites (adolescent and young adult ALL per year: <2 per year = 11%, ≥2 per year = 26%; *P*= .03).^13^ In contrast to these data, our NCORP study observed more older adolescents and young adults and adolescents and young adults at low-volume sites receiving pediatric-inspired therapy. Although NCORPs are community sites, they are also research focused; it is reasonable to conclude that the NCORP mission (bringing trials to the community) enhances their use of pediatric-inspired therapy, for instance, the NCORP is achieving its mission even if trial enrollment itself is variable. This may reflect an effect like the structure- and process-level influence that care at NCI-designated comprehensive cancer centers has on adolescent and young adult cancer outcomes.^12^^,^^40-44^

Considering these findings in the context of their limitations, there may be inherent differences between participating and nonparticipating practices; nevertheless, the sampling strategy optimized generalizability. Although retrospective studies by nature pose limitations, we optimized data reliability by reviewing supporting documentation. Because of high rates of guideline-concordant care, we were unable to adjust for other site characteristics.

In summary, facility-level characteristics associated with delivering guideline-concordant care (specifically pediatric-inspired therapy) in adolescent and young adult ALL were identified, but further work is crucial to understand facility-level predictors of adolescent and young adult trial enrollment. Although findings from NCORP care delivery research are as close as possible to the real-world community setting and presumed generalizable to community sites, true community practices treating adolescents and young adults presumably have lower guideline-concordant care and warrant strategies to enhance uptake of these regimens. Adult/internal medicine clinical facilities—of all sizes—need support most. Our findings suggest that strategies to enhance use of pediatric-inspired therapy across such settings could focus on enhancing communication between pediatric and adult counterparts (especially at adult/internal medicine and mixed clinical facilities) and facilitating adolescent and young adult–specific clinical pathways.

## Supplementary Material

pkaf033_Supplementary_Data

## Data Availability

The Children’s Oncology Group Data Sharing policy describes the release and use of COG individual subject data for use in research projects in accordance with National Clinical Trials Network (NCTN) Program and NCI Community Oncology Research Program (NCORP) Guidelines. Only data expressly released from the oversight of the relevant COG Data and Safety Monitoring Committee (DSMC) are available to be shared. Data sharing will ordinarily be considered only after the primary study manuscript is accepted for publication. For phase 3 studies, individual-level de-identified datasets that would be sufficient to reproduce results provided in a publication containing the primary study analysis can be requested from the NCTN/NCORP Data Archive at https://nctn-data-archive.nci.nih.gov/. Data are available to researchers who wish to analyze the data in secondary studies to enhance the public health benefit of the original work and agree to the terms and conditions of use. For nonphase 3 studies, data are available following the primary publication. An individual-level de-identified dataset containing the variables analyzed in the primary results paper can be expected to be available on request. Requests for access to COG protocol research data should be sent to datarequest@childrensoncologygroup.org. Data are available to researchers whose proposed analysis is found by COG to be feasible and of scientific merit and who agree to the terms and conditions of use. For all requests, no other study documents, including the protocol, will be made available, and no end date exists for requests. In addition to above, release of data collected in a clinical trial conducted under a binding collaborative agreement between COG or the NCI Cancer Therapy Evaluation Program and a pharmaceutical/biotechnology company must comply with the data sharing terms of the binding collaborative/contractual agreement and must receive the proper approvals.
