# Supplementary material for: Guideline-concordant treatment among adolescents and young adults with acute lymphoblastic leukemia
Source: JNCI Cancer Spectr. 2025 Apr 16;9(3):pkaf033. doi: 10.1093/jncics/pkaf033 (PMC12121638; doi:10.1093/jncics/pkaf033)

Supplementary Figure 1. CONSORT Diagram

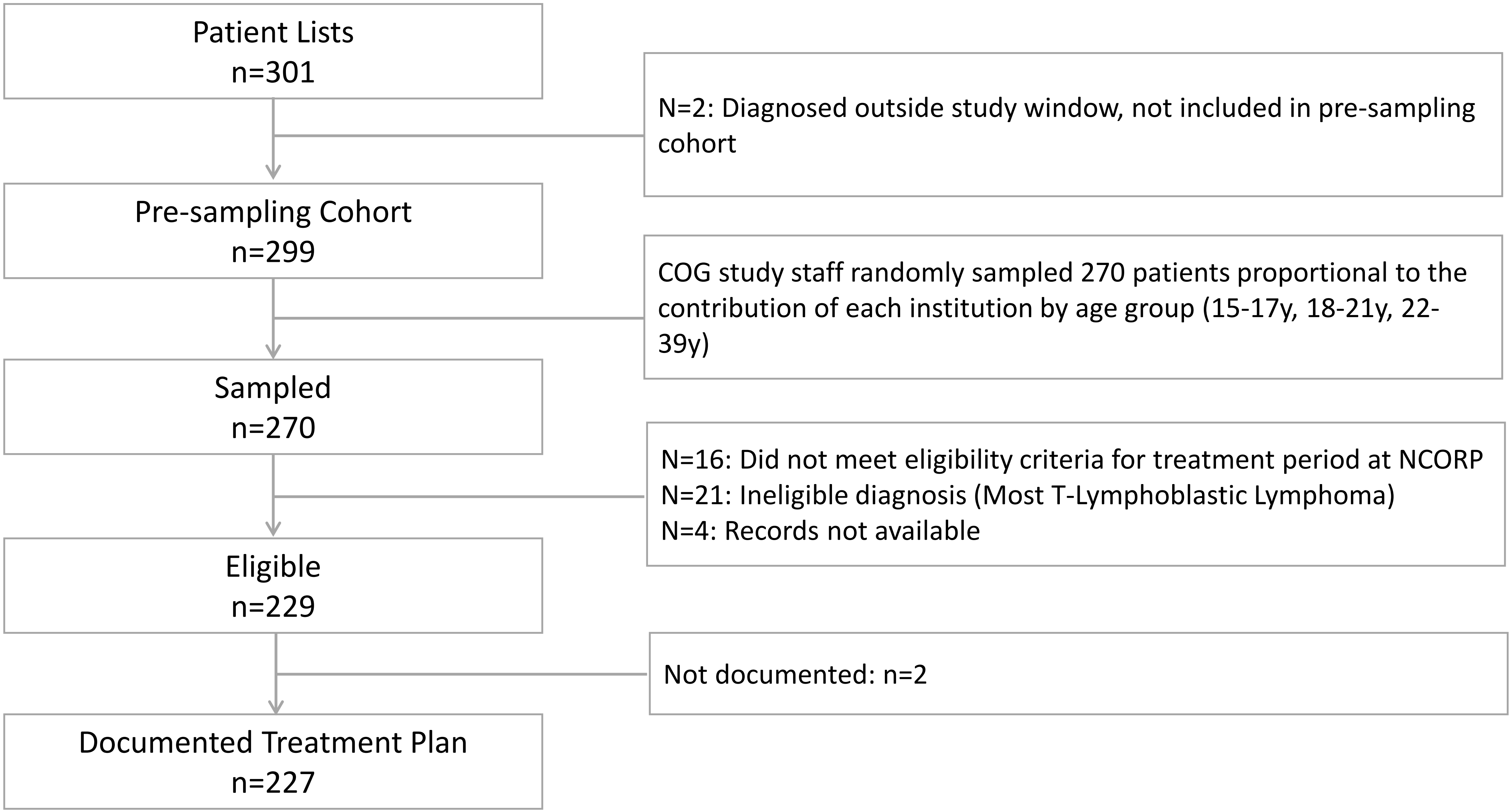

Supplementary Figure 2. Facility-level Characteristics of Clinical Facilities Where Adolescents and Young Adults with Acute Lymphoblastic Leukemia were Treated

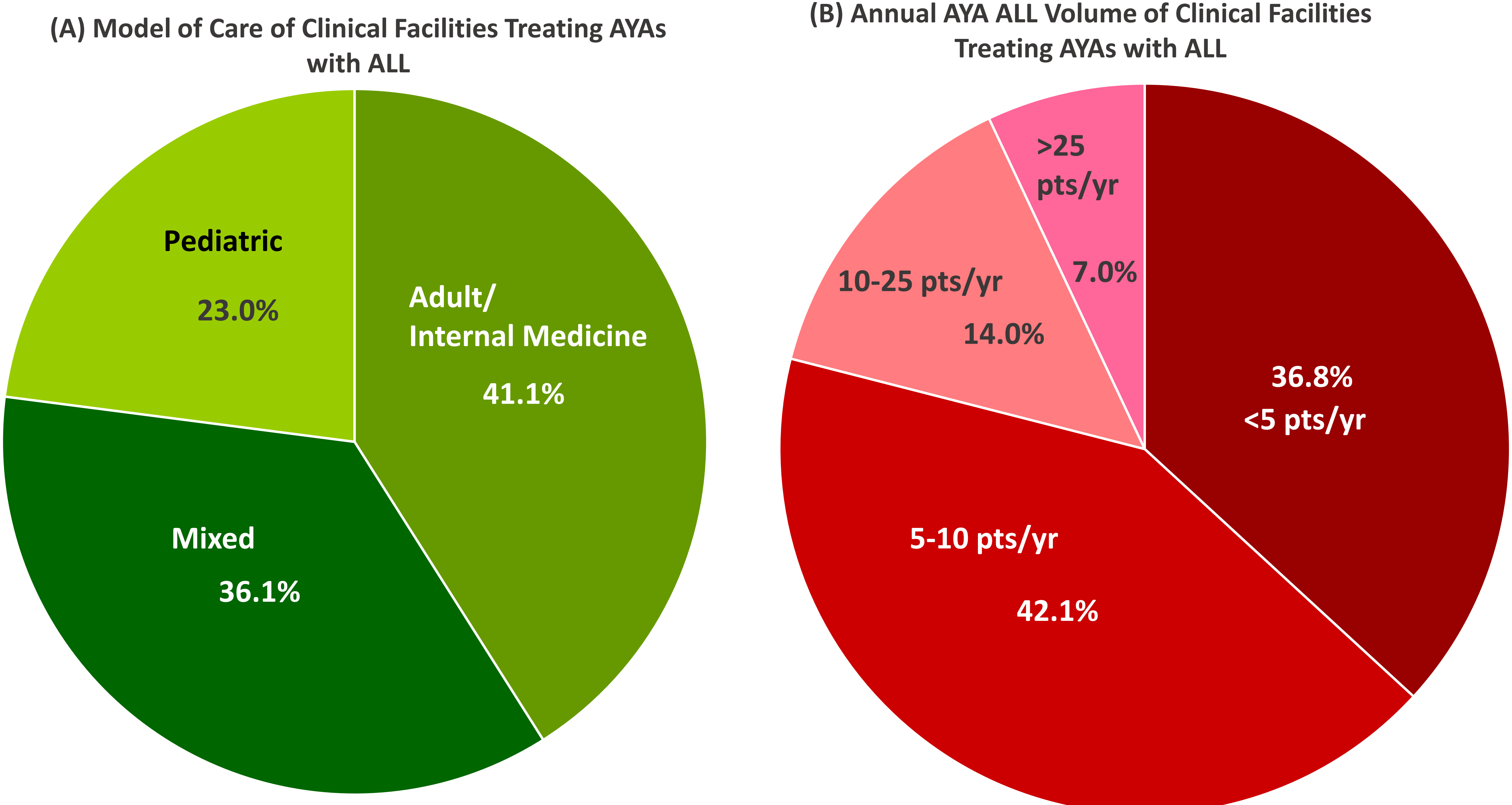

Supplement: pkaf033_Supplementary_Data [file pkaf033_supplementary_data.zip › 16N1CD_PtLevel_SUPPFig_FINAL_CLEANv3.pdf]
