# Supplementary material for: Guideline-concordant treatment among adolescents and young adults with acute lymphoblastic leukemia
Source: JNCI Cancer Spectr. 2025 Apr 16;9(3):pkaf033. doi: 10.1093/jncics/pkaf033 (PMC12121638; doi:10.1093/jncics/pkaf033)
Supplement: pkaf033_Supplementary_Data [file pkaf033_supplementary_data.zip › L16N1_Pt-levl_SUPPTables_2025Feb24_CLEAN.docx]

| Supplementary Table 1. Guideline Concordant Care: By Oncologist Specialty and ALL Subtype | | |
| --- | --- | --- |
| **ALL Subtype** | **Adult** | **Pediatric** |
| ***(% Guideline Concordant)***  ***[% On Study]*** | | |
| **Ph-neg** | 42 (62%; n = 69)  5 [7%; n = 69] | 90 (99%; n = 91)  58 [64%; n = 91] |
| **Ph-pos** | 13 (93%; n = 14)  0 (0%; n = 14) | 27 (100%; n = 27)  15 [56%; n = 27] |
| **T-ALL** | 11 (79%; n = 14)  1 [7%; n = 14] | 12 (100%; n = 12)  10 [83%; n = 21] |

**Supplementary Table 2. Odds of Delivery of Guideline-Concordant Treatment from unadjusted models:**

***At Adult Facilities Only***

|  | **OR** | **95%CI** |
| --- | --- | --- |
| **Facility Size** |  |  |
| <5 patients (ref) | -- | -- |
| 5-10 patients | 0.02 | 0.00-5061.80 |
| >10 patients | 0.04 | 0.00-89450.01 |
| **Age** | 0.87 | 0.63-1.19 |

**Supplementary Table 3. Guideline Concordant Care: By Race/Ethnicity and Age.**

| Age | Non-Hispanic white | All non-white races/ethnicities |
| --- | --- | --- |
|  | n (% guideline concordant; total n) | |
| 15-17 years | 28 (100%; n = 28) | 52 (100%; n = 52) |
| 18-21 years | 20 (87.0%; n = 23) | 41 (93.2%; n = 44) |
| 22-39 years | 22 (73.3%; n = 30) | 35 (70.0%; n = 50) |
